# The Adjuvant Therapy of Intravenous Laser Irradiation of Blood (ILIB) on Pain and Sleep Disturbance of Musculoskeletal Disorders

**DOI:** 10.3390/jpm12081333

**Published:** 2022-08-19

**Authors:** (Jimmy) Chun-Ming Fu, Nai-Kuang Wang, Yuan-Yang Cheng, Shin-Tsu Chang

**Affiliations:** 1 Department of Physical Medicine and Rehabilitation, Taichung Veterans General Hospital, Taichung 407219, Taiwan; 2 School of Medicine, National Yang Ming Chiao Tung University, Taipei 112304, Taiwan; 3 Department of Post-Baccalaureate Medicine, College of Medicine, National Chung Hsing University, Taichung 402010, Taiwan; 4 Department of Physical Medicine and Rehabilitation, Kaohsiung Veterans General Hospital, Kaohsiung 813414, Taiwan; 5 Department of Physical Medicine and Rehabilitation, Tri-Service General Hospital, School of Medicine, National Defense Medical Center, Taipei 110301, Taiwan

**Keywords:** intravenous laser irradiation of blood, musculoskeletal pain, sleep quality, helium-neon laser light

## Abstract

(1) Background: Musculoskeletal pain is both intractable and irritating. Intravenous Laser Irradiation of Blood (ILIB) therapy has been used as pain control treatment for this condition. However, there remains a lack of clear evidence regarding ILIB on pain control. This study aimed to reveal the result of changes in patient pain intensity after receiving ILIB therapy. (2) Methods: We conducted a retrospective analysis of pain scores and sleep quality from 76 patients diagnosed with musculoskeletal disease who received three courses of ILIB therapy. Each course included ten sessions of ILIB treatment over ten consecutive days. During ILIB therapy, patients were inserted with a laser fiber optic needle which irradiated blood cells via a 632.8 nm Helium-Neon laser light over a period of 60 min. Pain scores were evaluated using the Visual Analogue Scale (VAS), whereas sleep quality was assessed by the Pittsburgh Sleep Quality Index (PSQI). These scores would be recorded both before and after each ILIB treatment course. (3) Results: The mean of all patients’ initial VAS score was 5.35. After completing three courses of ILIB treatment, the mean VAS score decreased to 2.2, which indicated a significant reduction in pain intensity. Additionally, patients experienced sleep quality improvement levels from PSQI 8.97 to 5.53 upon completion of three courses of ILIB treatment. (4) Conclusions: Intravenous Laser Irradiation of Blood (ILIB) resulted in a positive pain control effect on patients with musculoskeletal disease, especially for those with moderate to severe pain intensity (initial VAS score >4). Additionally, patients experienced better sleep quality as a result of their pain relief after receiving ILIB treatment.

## 1. Introduction

Musculoskeletal disorders are troublesome conditions which involve pain, sleep disturbance, fatigue, and Range of Movement (ROM) limitations. There are approximately 1.7 billion people worldwide suffering from musculoskeletal disorders, which further lead to poor life quality, decreased work efficiency, and even feelings of frustration [1]. A rehabilitation program plays an important role during the early stages of treatment. Modalities such as thermal therapy, cryotherapy, hydrotherapy, electrotherapy, and lower-level laser therapy have been widely used for treating pain resulting from musculoskeletal disorders [2,3]. Thanks to these mature techniques, musculoskeletal pain can be relieved while one’s social well-being improves and social costs decrease. Nevertheless, researchers are still committed to the study of new treatment options for the purposes of pain control.

Currently, Intravenous Laser Irradiation of Blood (ILIB) therapy is being applied as a means of adjuvant pain control for conditions such as diabetes neuropathy, fibromyalgia, and arthritis [4,5,6]. ILIB is a user-friendly technique performed by inserting a needle into the vein to help purify the circulation system. A Helium-Neon laser with the wavelength of 632.8 nm can irradiate whole blood cells and benefit blood rheology [7]. According to previous studies, a lower powered laser can enhance red blood cell oxygen and erythrocyte deformability [8,9,10,11]. Platelet aggregation and adhesion are also inhibited after photodynamic reactions and therefore blood viscosity can decrease [9,12]. Moreover, immunity can be modulated by increasing the mitochondrial function of white blood cells [11,13,14]. The enhancement of total antioxidant capacity was also recorded to prevent excessive oxidative stress attack and promote healing of damaged cells [13].

ILIB was first applied by Russian scientists in 1981. The technique was initially introduced to treat cardiovascular disease because it facilitates blood circulation and refines blood cell function [7]. Afterwards, ILIB was mainly used in both post-stroke adjuvant therapy and coronary artery restenosis prevention [9,12]. Upon further development, researchers also applied the technique to spinal cord injuries, type 2 diabetes mellitus, arthritis, and multiple sclerosis [15].

Apart from these applications, ILIB can also benefit organ systems by regulating immunity, increasing blood cell oxygen capacity, and facilitating blood flow, so it seems to provide tissue repair while bringing about the effects of pain control. Some reports have mentioned its ability for pain control when treating fibromyalgia and diabetic neuropathy [4,6,7]. However, few studies have actually been conducted which confirm the effects of pain decrease through ILIB treatment, with those results being obtained from only a relatively small study population. Therefore, further results from studies surrounding ILIB treatment and pain control are still needed. This study aimed to confirm the effect of ILIB on pain by comparing pain scores quantitatively both before and after intravenous laser irradiation of blood therapy.

## 2. Materials and Methods

### 2.1. Study Design and Setting

**Figure 1:**
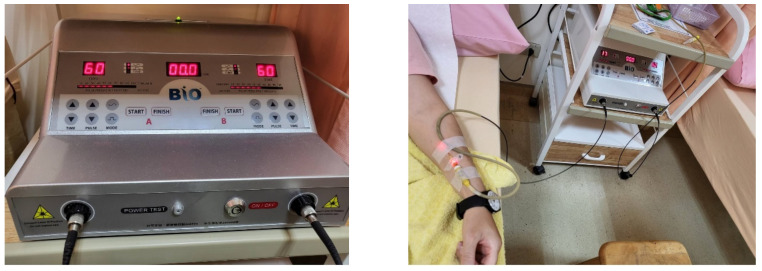
ILIB equipment (Left) and settings during ILIB treatment (Right).

This research involves a retrospective analysis, with the data being retrieved from patients receiving ILIB treatment during the period of August 2017 to August 2021 in the Department of Physical Medicine and Rehabilitation, Taichung Veterans General Hospital, Taiwan. All patients would receive ten sessions of ILIB treatment over ten consecutive days for one course of treatment. Patients would receive at least three courses of treatment. During ILIB treatment, a laser fiber optic needle was inserted into the blood vessel, where a 632.8 nm Helium-Neon laser light would directly irradiate blood cells via the BIO-ILIB He-Ne Laser machine manufactured in Taiwan (Figure 1). The laser output level was set at 6mW, with the procedure maintained for 60 min per treatment. The patients would be recorded with a pain score and sleep quality level before and after ILIB treatment. According to our chart review, the consensus end point of terminating the ILIB treatment is when patients feel pain subside or when the pain is acceptable and does not interfere with daily life. The study was approved by our Institutional Review Board.

### 2.2. Participants

**Figure 2:**
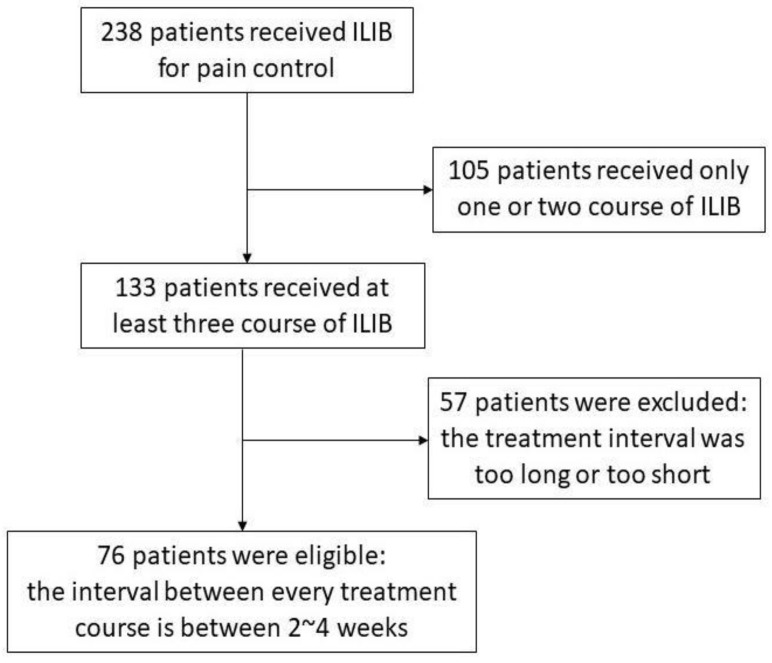
Patient inclusion flow chart.

Patients were included when they received ILIB treatment for the purpose of musculoskeletal pain control. The musculoskeletal disorders included were spondylopathies, enthesitis, tendinitis, periostitis, osteoarthritis (OA), and others. Due to the retrospective nature of the study, we set some inclusion criteria in order to unify the participants’ characteristics: (1) Patients suffered from pain for at least 3 months and should have completed at least three courses of ILIB treatment. (2) The interval between each treatment course should have been between 2~4 weeks. (3) Patients had more multiple sites of pain, rather than a single site. (4) Patients either with or without regular painkillers usage all could be included in the study. The participants did not have to take other conservative treatments first and then receive ILIB treatment only when other methods failed. (5) The patients were allowed to continue refilling their prescriptions of analgesic drugs as scheduled, but the analgesic doses were not to be titrated up during ILIB treatment (Figure 2). That is to say, if patients were to be given an increased dose of an analgesic drug, it may mask the effect of ILIB pain control, causing participants to be excluded. In addition, although there has been no report about obvious adverse events of ILIB treatment so far, patients with recent hemorrhage history, photosensitivity, infection status, or pregnancy were excluded to receive ILIB treatment, according to our routine clinical practice [6,16].

### 2.3. Study Variables

The pain evaluation was measured by the Visual Analog Scale (VAS), which is a reliable tool for recording pain intensity at a range from 0 to 10 points [17]. We would assess pre-test VAS prior to ILIB treatment as a pain intensity baseline and subsequently evaluate the post-test VAS shortly after the end of a course of ILIB treatment. Furthermore, we also recorded patients’ sleep quality through use of the PSQI score (Pittsburgh Sleep Quality Index), which is a questionnaire composed of 7 components. Each component ranges from 0 to 3 points, with a total score of 21 points being possible [18]. A higher score indicates a worse quality of sleep.

### 2.4. Identification Characteristics for ILIB Treatment Optimization

We considered that different blood composition and biochemistry profile may affect the outcome of ILIB; patients were then divided into two groups according to blood characteristics, for example: an anemia group (male hemoglobin <13 g/dL or female hemoglobin <12 g/dL) and a non-anemia group (male Hb >13 g/dL or female Hb >12 g/dL). Additionally, we also grouped these participants according to platelet counts (<200,000/μL; >200,000/μL), hsCRP levels (<0.3 mg/dL; >0.3 mg/dL), HbA1c levels (<6.5%; ≥6.5%), and gender (male; female). These characteristics would be analyzed through pain score results after receiving ILIB treatment.

### 2.5. Statistical Analysis

The Wilcoxon signed-rank test was performed because participants’ VAS scores did not reach normal distribution according to the Kolmogorov–Smirnov test. Nonparametric statistics can present more conservative results [19]. The pre-test VAS and PSQI scores were compared with the post-test VAS and PSQI scores after each course of treatment. In addition, the pre-test score among each course would be compared so as to evaluate the differences among each course of ILIB outcomes. We also analyzed the pain relief effect of different diseases individually. When the participants’ numbers of diseases reached five or more (*n* ≥ 5), we performed Wilcoxon signed-rank test to evaluate the significance of VAS difference after completing three courses of ILIB treatment. If the participants’ numbers were less than five (*n* < 5), we simply assessed descriptive statistics. A statistical significance level was set at *p* < 0.05. All analyses were performed with SPSS, Ver.20, IBM, (New York, NY, USA).

## 3. Results

### 3.1. Patient’s Profile

Seventy-six patients met the selection criteria, with ages ranging from 30 to 86 years, with a mean age of 57.7 years old. Twenty-four males and fifty-two females were involved in the study. The main diagnosis of each patient was listed as Table 1. In terms of side effects, bruise and needling difficulty was recorded in three ILIB treatments of different participants. No serious adverse effect which needed medical intervention was documented in the ILIB treatment.

### 3.2. Total Participants’ VAS Score Trend

**Figure 3:**
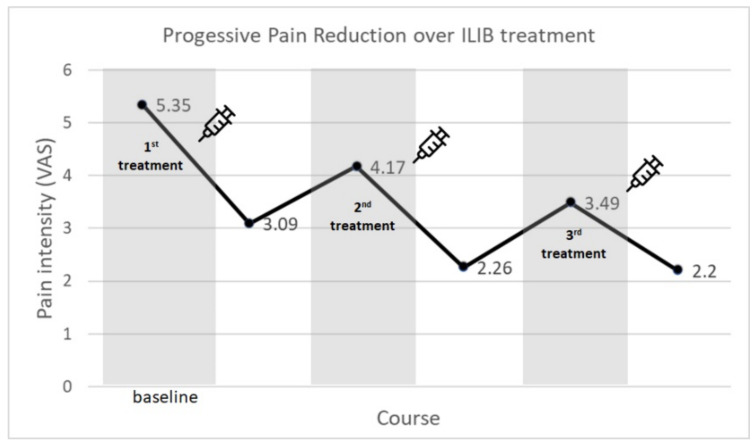
ILIB treatment showed the pain reduction effect over three courses of treatment. (VAS scale: 0–10).

In the 76 patients diagnosed with musculoskeletal disease, the initial mean VAS score was 5.35 prior to receiving any ILIB treatment. After completing the first course of ILIB, the VAS score decreased to 3.09 points. Pain intensity decreased with statistical significance after the first course of ILIB treatment (Table 2). Similarly, the mean VAS scale before and after the second course of treatment was 4.17 and 2.26, respectively, with the difference also reaching statistical significance. During the third course of ILIB, the patients had 3.49 points on VAS prior to treatment and 2.20 at the end of treatment. These results concluded that the patients’ pain intensity would experience immediate relief effectively during the ILIB treatments. In addition, the pain intensity levels throughout every course were also compared. The pain intensity levels prior to the first course (VAS: 5.35), second course (VAS: 4.17), and third course (VAS: 3.49) of ILIB treatment showed significant differences (*p* value < 0.001). This indicated that participants experienced a pain decrescendo trend during the three sessions of ILIB treatment courses (Figure 3).

Table 3 revealed the effect of ILIB treatment in patients with different diseases. Patients suffering from spinal enthesopathy, periostitis, radiculopathy, and polyneuropathy had significantly decreased pain intensity after completing ILIB treatment. Only patients with osteoarthritis did not have significant improvement; nonetheless, the VAS score of these patients still showed a downward trend after receiving treatments (VAS: 6.17 decreased to 4.83). As for those diseases with patients number less than five (*n* < 5), the descriptive statistic results showed all diseases, but tendinitis had the trends of pain intensity decrease. As stated above, Table 3 suggests that most of the various musculoskeletal diseases achieved effective pain relief after completing three courses of ILIB treatment.

### 3.3. Identification Characteristics for ILIB Treatment Optimization

**Figure 4:**
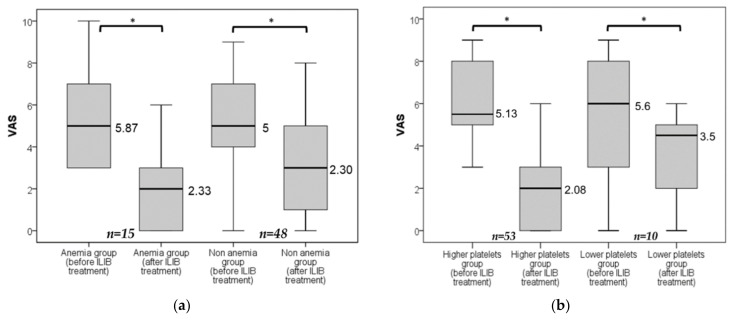

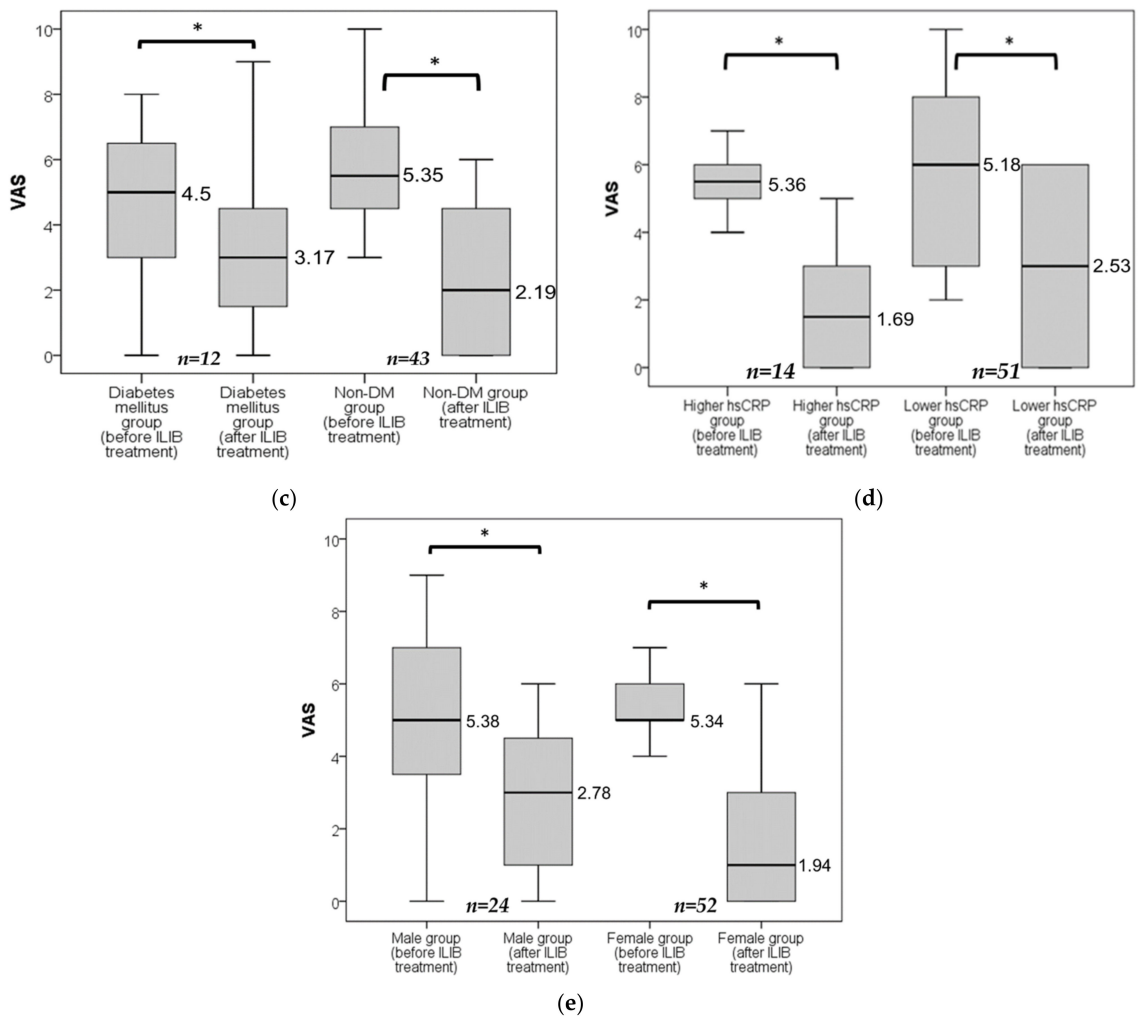
(**a**–**e**) The comparison between the initial VAS and the final VAS after completing three courses of ILIB treatment in different groups. Anemia group, male participants with Hb <13 g/dL or female with Hb <12 g/dL; Lower platelets group, participants with platelet counts <200,000/μL; Diabetes mellitus group, participants with HbA1c ≥6.5%; Higher hsCRP group, hsCRP level >0.3 mg/dL; * All groups showed a significant decrease in VAS value (*p* < 0.001) when participants had completed the three courses of ILIB treatment.

We allocated patients to two groups according to blood hemoglobin status, as shown in Figure 4a. The results revealed that whether the patient is anemic (male Hb <13, female Hb <12) or not, ILIB treatments for pain relief are equally effective. In the same way, we divided participants based on blood platelet count, blood sugar, blood hsCRP, and gender, where all the groupings showed a statistically significant decrease on VAS after completing ILIB treatment courses (Figure 4b–e). This finding indicated that the above characteristics did not interfere with the pain control effect in the populations.

### 3.4. Pain Control Effect in the Groups of Different Pain Intensity

To evaluate the pain control effect in the groups of different pain severity, we divided participants into three groups according to their initial VAS scale before receiving the ILIB treatment (Mild pain group: 0 < VAS ≤ 3; Moderate pain group: 3 < VAS ≤ 7; Severe pain group, 7 < VAS ≤ 10). As described in Table 4, the moderate pain group and the severe pain group had the significant VAS scale improvement after completing three courses of ILIB treatment. However, the mild pain severity group showed no statically significant VAS difference after completing the ILIB treatment (p value = 0.352). What is more, the moderate pain group obtained a lower VAS score (1.65) than that of the mild pain group (1.88) after completing three courses of ILIB treatment. In consequence, the ILIB treatment brought a greater benefit to the moderate and severe pain groups, compared with the mild pain group.

### 3.5. Participants’ Sleep Quality

**Figure 5:**
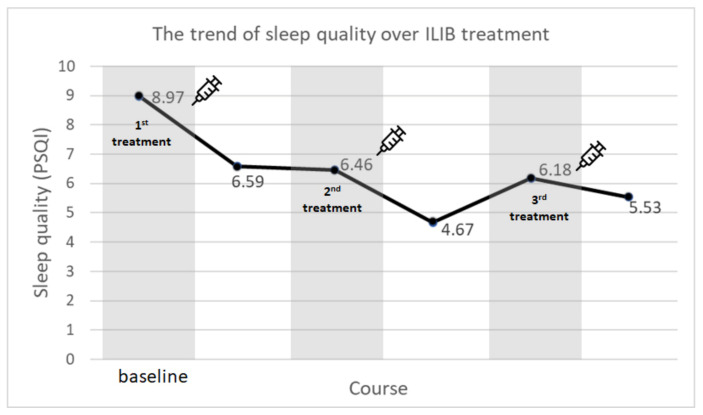
Although sleep quality showed temporary improvement during any course of treatment, the ILIB could not provide further improvement of sleep quality after patients received the second and third courses of treatment.

The PSQI score (sleep quality) for patients improved significantly from 8.97 to 6.59 after completing the first course of ILIB treatment (Table 5). Sleep quality improvement was also noted when receiving the second or third episode of ILIB treatment. Patients indeed obtained short-term sleep improvement whenever they received each episode of treatment. However, there was no statistically significant difference between patient PSQI scores before the second ILIB course (PSQI = 6.46) and that performed before the third ILIB course (PSQI = 6.18). This revealed that patients were unable to receive any further accumulative sleep improvement effects after completing the second and third courses of ILIB treatment. In summary, ILIB treatment can provide short-acting sleep quality enhancement but cannot offer an accumulative sleep quality effect (Figure 5).

### 3.6. The Safety of ILIB Treatment

When we review the 76 patients’ medical records, there was no record about obvious adverse effects of ILIB treatment. Consequently, the intravenous laser irradiation of blood (ILIB) treatment can be regarded as a safe tool for musculoskeletal pain control.

### 3.7. Participants Who Only Received 1 or 2 Courses of ILIB

As described in Figure 1, there were 105 patients only receiving 1 or 2 courses of ILIB treatment because they obtained obvious pain control and they thought there was no need to have more courses of treatment. Therefore, they terminate subsequent treatments by themselves. Although we suggest patients receive three courses of ILIB treatment, there is no mandatory provision, and these patients are free to choose how many courses of treatment they want to have. Therefore, most patients discontinued the subsequent treatment (that is to say, only received 1~2 courses of treatment) as soon as they were satisfied with the ILIB pain control effects. When we performed the post hoc analysis, it revealed that the mean of the initial VAS score of the 105 patients was 5.07, and the VAS score had a statically significant decline to 2.67 points (*p* value < 0.001) after patients completed the first course of ILIB treatment. Similarly, after the second course of ILIB treatment, the mean of the VAS score improved from 3.08 to 1.92 in these patients (*p* value < 0.001). The results explained that patients receiving only one or two courses of ILIB also received a satisfying pain control result.

## 4. Discussion

This study revealed that patients could obtain musculoskeletal pain improvement after receiving three courses of ILIB therapy, especially for moderate to severe pain intensity groups (initial VAS score >4). The results also confirm that ILIB has an accumulative effect on pain control. ILIB treatment provides a novel and promising pain control option different from other conventional medical treatments, which include physical therapy, splints, acupuncture, steroid injections, and drug treatment. However, ingesting analgesic drugs such as NSAID or opioids may result in side effects such as gastrointestinal tract upset, renal disturbance, and cardiovascular events [20]. Physical therapy options, such as thermal therapy, therapeutic massage, and electrotherapy are inconvenient when patients have several painful sites or tender points to treat. As for steroid injections, they must rely on highly operator-dependent sonography skills which manipulators must spend a lot of time learning [21]. Hence, intravenous laser irradiation of blood treatment provides an additional safe, convenient, and effective way to relieve musculoskeletal pain. Patients receiving ILIB treatment simply need to accept a laser fiber optic needle injection and lie down for 60 min as they wait to purify their blood circulation. It is a reliable and user-friendly treatment worthy of being applied in patients with musculoskeletal pain.

The participants included in our study all had more than one site of pain, and ILIB successfully brought the multi-site pain control effects according to our medical records review. We believe ILIB could bring systemic pain control effects, rather than just local side analgesic results. This is the competitive advantage which local steroid injection, therapeutic massage, or local lower-level-laser-therapy (LLLT) lack. The reasons why ILIB could bring systemic pain control effect are described as follows.

There are multi-faceted explanations about ILIB therapy on pain control, including blood rheology, metabolism regulation, anti-inflammation property, and nerve system stimulation. First of all, ILIB treatment can reduce blood viscosity, inhibit thrombosis, enhance red blood cell oxygen capacity, therefore improving blood circulation. In certain previous studies, it has been disclosed that ILIB can increase cerebral blood flow [22,23,24]. By supplying the brain and muscle tissue with additional oxygen and nutrition, these refined blood cells may create a proper microenvironment for repairing damaged tissue [9,12]. Additionally, some previous studies have also mentioned that ILIB treatment could help eliminate toxins, such as free radicals and peroxides, and increase superoxide dismutase (SOD), an antioxidant agent [12]. Therefore, ILIB can prevent damage from an oxidative stress attack while promoting the healing of musculoskeletal tissues [13]. The light of ILIB also stimulates the mitochondrial respiratory chain to enhance adenosine triphosphate synthesis (ATP) synthesis, which is needed by muscle and nervous tissue metabolism [7,14,25].

IL-1α, IL-1β, and IL6 are pro-inflammatory cytokines which exacerbate acute or chronic inflammatory diseases. In an animal model, intravenous laser energy can lower the level of interleukins and facilitate the anti-inflammation effect [26]. Furthermore, ILIB can accelerate the growth of nerve fiber microtubules and neuron axon motion as a result of the metabolic process and improve the microcirculation of innervated organs [6,12]. These would protect patients’ neuromuscular system. Based upon the characteristics previously mentioned, we can therefore reasonably explain the mechanisms of ILIB treatment on musculoskeletal pain control. What is more, a higher level of inflammation and tissue damage are related to the severity of musculoskeletal diseases, and therefore ILIB could bring more anti-inflammation and tissue repair effects on patients with moderate to severe pain, thus bringing more pain control results, as shown in Table 5.

As stated above, patients with inflammation conditions (e.g., spinal enthesopathy, periostitis, or polymyositis) or degenerative disorders (e.g., osteoarthritis, DJD, or spondylolisthesis) may have an anti-inflammation effect and be protected from an oxidative stress attack when receiving ILIB treatment, which decreases pain intensity. As for polyneuropathy or spinal cord injury, ILIB may facilitate nerve fiber growth and neuron axon regeneration, leading to pain intensity improvement. In some acute pain cases, such as bone fracture, we could not explain that ILIB therapy is the main cause of pain relief, because the musculoskeletal tissue can also naturally heal itself to relieve pain, but we could view ILIB as a pain relief adjuvant therapy because it could promote nutrition and oxygen from blood flow and enhance the ATP synthesis, which facilitate the microenvironment for repairing damaged musculoskeletal tissue.

OA patients experienced pain relief without statistical significance. However, we still can see the downward trend of VAS score during three sessions of ILIB. The *p* value is 0.066 and the reason that failed to reach significant level is mainly due to the small sample population. The reason for the small sample size is because the mainstream treatment of degenerative arthritis is rehabilitation, hyaluronic acid, and regenerative injection, which generally achieve good results. The patients who received ILIB treatment are those recalcitrant to the OA routine therapy and thus contribute to both the small number of participants and the insignificant effect. Given the pain relief of different musculoskeletal diseases described in Table 3, we can obtain reasonable explanations.

In our study, it was revealed that patients were able to acquire accumulative pain improvement when receiving three courses of ILIB treatment. Regardless of whether the patient had anemia or not, had higher or fewer platelets, was diagnosed with or without diabetes mellitus, or had a higher or lower inflammation level, ILIB therapy could provide pain control to these participants (Figure 3). Moreover, patient who received only 1–2 courses of ILIB can also receive some degree of pain reduction according to our post hoc analysis; very few of the participants received more than three courses of ILIB treatment for the purpose of pain control; therefore, we did not perform further analysis.

According to previous studies, a change of three points or more in PSQI score was viewed as a minimal clinically important difference (MCDI) [27,28]. When patients’ PSQI score improved by at least three points, they would actually feel a noticeable improvement in their sleep quality. In our results, there were 44% of patients showing PSQI improvement by at least three points after completing three courses of ILIB treatment. Therefore, from a clinical perspective, ILIB indeed benefitted part of the patients’ sleep quality.

The ILIB therapy provides the short-term effect of sleep quality promotion after treatment, although the accumulative effect on sleep quality needs to be further investigated. We assume that sleep quality is enhanced as a result of pain improvement in patients after ILIB treatment [29,30]. Moreover, better sleep quality may in turn provide a more suitable microenvironment for tissue repair [31]. To maintain physiological function, a body’s metabolism during waking hours initiates energy burning and the release of free radicals, which leads to cell injury [32]. When humans enter the sleep cycle, the metabolic rate and body temperature decreases [33,34]. Damaged tissues would then undergo physiological repair during Non-rapid Eye Movement sleep stage 3 (NREM-3) [35,36]. Additionally, growth hormone secretion during sleep also promotes bone and muscle tissue repair [37,38]. Therefore, the bidirectional relationship between the effects of ILIB pain control and sleep quality improvement builds up a virtuous circle. The analgesic effect of ILIB therapy can also result in better sleep quality, as sleep then in turn promotes musculoskeletal tissue repair.

Although ILIB has been successfully implemented as a pain control treatment tool previously, there still remains a lack of clear evidence and reference sources. The ILIB treatment method for pain control is viewed as an off-label use tool. To the best of our knowledge, this is the first retrospective study to confirm the results of musculoskeletal disease pain control from ILIB treatment. However, this study has some limitations which are worthy of further research. We only adopted three courses of ILIB treatment, so the effect of additional courses of ILIB treatment requires further research. Furthermore, no control group was used to compare the results because there were no proper data from our medical records. Therefore, it is worth performing a further prospective study or randomized controlled trial to evaluate ILIB treatment effects. More importantly, there was no long-term follow up on the effects of pain control after ILIB treatments in this study, with our team knowing only the immediate analgesic effects after receiving ILIB treatment. How long the analgesic effects of ILIB treatment can be maintained is also an issue for discussion. Finally, owing to the limited participant numbers involved in this study, we did not conduct ILIB research on the specific disease but rather broadly included all skeletomuscular diseases (Table 1). In contrast, we placed more focus on the effects that ILIB pain control had on general musculoskeletal pain.

## 5. Conclusions

In our study, Intravenous Laser Irradiation of Blood (ILIB) therapy showed pain intensity reduction results on musculoskeletal diseases. As for moderate to severe pain intensity patients, they would obtain more pain control effects from ILIB therapy. By improving blood circulation, promoting metabolism, and scavenging free radicals, the treatment may facilitate musculoskeletal tissue repair as well as pain control. In our study, ILIB has not aimed to outperform other pain control treatments. We did not directly compare the effect of ILIB therapy with other treatments, and we just objectively analyzed the pain reduction results of ILIB therapy. Even so, our study actually revealed that ILIB had positive pain improvement results on patients with musculoskeletal disease. In conclusion, ILIB therapy provides a safe and promising choice for patients experiencing musculoskeletal pain.

## Figures and Tables

**Table 1:** The disease classification of patients who received Intravenous Laser Irradiation of Blood treatment.

Disease	Number (*n*)
Spinal enthesopathy	19
Periostitis	11
Radiculopathy (HIVD)	8
Osteoarthritis	6
Polyneuropathy	5
Degenerative joint disease	4
Myofascial pain syndrome	4
Spondylolisthesis	4
Tendinitis	3
Frozen shoulder	3
Other diseases ^a^	8

^a^ Other diseases included Polymyositis, Diffuse idiopathic skeletal hyperostosis, Systemic Lupus erythematosus, Reflex sympathetic dystrophy, Spinal cord injury, Fibrositis, Fracture of femoral bone medial condyle, Rib fracture.

**Table 2:** The Visual Analogue Scale (VAS) of patients with musculoskeletal pain before and after receiving ILIB treatment.

	Before Treatment		After Treatment		*p* Value
	*Mean*	*SD*	*Mean*	*SD*	
1st course of ILIB	5.35	2.12	3.09	1.98	<0.001
2nd course of ILIB	4.17	2.34	2.26	2.11	<0.001
3rd course of ILIB	3.49	2.44	2.20	2.33	<0.001

Each course of ILIB includes ten sessions of ILIB treatment over ten consecutive days. These participants received three courses of treatment, with the interval between every treatment. course being approximately 2~4 weeks. Data mentioned above were collected from all of the 76 patients.

**Table 3:** The pain intensity change of different disease types.

	VAS Score			
	Before All ILIB Treatments Mean ± SEM	After All ILIB Treatments Mean ± SEM	*p* Value	*n*
Spinal enthesopathy	5.74 ± 1.94	1.44 ± 1.98	0.001 *	19
Periostitis	4.36 ± 1.20	2.00 ± 1.34	0.007 *	11
Radiculopathy (HIVD)	6.00 ± 1.51	1.63 ± 2.07	0.017 *	8
Osteoarthritis	6.17 ± 2.23	4.83 ± 3.25	0.066	6
Polyneuropathy	6.00 ± 3.16	2.40 ± 2.30	0.042 *	5

* Other conditions had fewer than five individuals.

**Table 4:** The Visual Analogue Scale (VAS) change of the groups of different initial pain intensity.

Pain Intensity	Before Treatment		After Treatment			*p* Value	*n*
	*Mean*	*SD*	*Mean*	*SD*	*Mean Difference*		
mild	2.35	1.06	1.88	2.32	0.47	0.352	17
moderate	5.52	0.88	1.65	1.93	3.87	<0.001	44
severe	8.27	0.59	4.13	2.56	4.14	0.001	15

Mild pain group, 0 < initial VAS ≤ 3; Moderate pain group, 3 < initial VAS ≤ 7; Severe pain group, 7 < initial VAS ≤ 10. The mean difference was ‘‘the difference between the mean VAS value in groups before and after completing three courses of ILIB treatment’’.

**Table 5:** The Pittsburgh Sleep Quality Index (PSQI) of patients with musculoskeletal disease before and after receiving ILIB treatment.

	Before Treatment		After Treatment		*p* Value
	*Mean*	*SD*	*Mean*	*SD*	
1st course of ILIB	8.97	4.18	6.59	4.63	<0.001
2nd course of ILIB	6.46	4.19	4.67	3.70	<0.001
3rd course of ILIB	6.18	4.58	5.53	4.29	0.011

Each course of ILIB includes ten sessions of ILIB treatment over ten consecutive days. These participants received at least three courses of treatment, with the interval between every treatment course being approximately 2~4 weeks.

## Data Availability

Not applicable.
